# Elevated Native T1 Values in the Remote Myocardium Supplied by Obstructive Non-Infarct Related Coronary Arteries in Post-STEMI Cardiac Magnetic Resonance

**DOI:** 10.1159/000528143

**Published:** 2022-11-22

**Authors:** Yafim Brodov, Shlomi Matetzky, Eli Konen, Mattia Di Segni, Anan Younis, Ayas Massalha, Anat Berkovitch, Arkadi Beytelman, Fernando Chernomordik, Orly Goitein

**Affiliations:** ^a^From the Heart Institute, Sheba Medical Center, Tel Hashomer, Sackler Faculty of Medicine, Tel-Aviv University, Tel Aviv, Israel; ^b^Department of Diagnostic Imaging, Sheba Medical Center, Tel Hashomer, Sackler Faculty of Medicine, Tel-Aviv University, Tel Aviv, Israel

**Keywords:** Cardiac magnetic resonance, Elevated native T1, STEMI, Non-infarcted myocardium, Obstructive non-infarct-related artery

## Abstract

**Introduction:**

Native T1 mapping values are elevated in acutely injured myocardium. We sought to study whether native T1 values, in the non-infarct related myocardial territories, might differ when supplied by obstructive or nonobstructive coronary arteries.

**Methods:**

Consecutive patients (*N* = 60, mean age 59 years) with the first STEMI following primary percutaneous coronary intervention, underwent cardiac magnetic resonance within 5 ± 2 days. A retrospective review of coronary angiography reports classified coronary arteries as infarct-related coronary artery (IRA) and non-IRA. Obstructive coronary artery disease (CAD) was defined as stenosis ≥50%. Native T1 values were presented using a 16-segment AHA model according to the three main coronary territories: left anterior descending (LAD), left circumflex (LCX), and right coronary artery (RCA).

**Results:**

The cutoff native T1 value for predicting obstructive non-IRA LAD was 1,309 msec with a sensitivity and specificity of 67% and 82%, respectively (AUC 0.76, 95% CI: 0.57–0.95, *p* = 0.04)*.* The cutoff native T1 value for predicting obstructive non-IRA RCA was 1,302 msec with a sensitivity and specificity of 83% and 55%, respectively (AUC 0.7, 95% CI: 0.52–0.87, *p* = 0.05). Logistic regression model adjusted for age and infarct size demonstrated that native T1 was an independent predictor for the obstructive non-IRA LAD (OR 4.65; 1.32–26.96, *p* = 0.05) and RCA (OR 3.70; 1.44–16.35, *p* = 0.03).

**Conclusion:**

Elevated native T1 values are independent predictors of obstructive non-IRA in STEMI patients. These results suggest the presence of concomitant remote myocardial impairment in the non-infarct territories with obstructive CAD.

## Introduction

Cardiac magnetic resonance (CMR) native T1 mapping allows accurate noninvasive quantitation of the myocardial damage by measuring the longitudinal myocardial relaxation [1]. Elevated native T1 values in the infarcted myocardial segments precisely quantify the myocardial injury [2, 3, 4]. The differentiation between reversible and irreversible myocardial injury, the relation to infarct transmurality, and adverse outcomes were all correlated with increased native T1 values [2, 3]. Acute versus chronic myocardial infarction, in STEMI patients, is reliably characterized using the threshold based on native T1 maps [4]. However, the data regarding non-infarct, remote myocardial segments and their impairment during STEMI remain controversial. We hypothesized that native T1 values in the remote non-infarct myocardial territories may differ if supplied by obstructive or nonobstructive coronary arteries. The aim of this study was to quantify and compare the early post-STEMI native T1 values of myocardial territories supplied by obstructive and nonobstructive coronary arteries in the non-infarct territories.

## Materials and Methods

### Study Design

Consecutive patients (*N* = 60) with the first STEMI between January 2017 and October 2018 were enrolled in a single-center study (Heart Institute, Sheba Medical Center). STEMI was defined as per current guidelines, and clinical management was undertaken according to contemporary practice [5, 6]. Briefly, the infarct-related coronary artery (IRA) in STEMI patients was confirmed by a dedicated analysis combining ST segment elevation by ECG, wall motion abnormalities on echocardiography, as well as TIMI coronary flow <3 during invasive angiography. In cases of TIMI flow = 3 in IRA, we were still confident to determine IRA basing on the presence of severe coronary stenosis, corresponding ST segment elevation, and wall motion abnormalities. In our cohort, normal TIMI flow = 3 was found in all non-IRA vessels.

All patients underwent invasive coronary angiography, primary percutaneous coronary intervention (PCI), and stenting of the IRA within 6 h of symptom onset. The decision to perform simultaneous non-IRA revascularization was at the discretion of the performing invasive cardiologist. Exclusion criteria included prior history of myocardial infarction, coronary artery bypass graft surgery, significant valvular disease, known cardiomyopathy, hemodynamic instability, implanted pacemakers or defibrillators, and claustrophobia. The control group consisted of 21 otherwise healthy volunteers (mean age 44 ± 10 years old, 52% female) who underwent a dedicated CMR study in order to provide normal reference values of myocardial native T1, as part of 3 Tesla MRI scanner calibration. Written informed consent to perform CMR was signed by all subjects, according to the local institutional review board requirements. A binary definition of obstructive and nonobstructive coronary artery disease (CAD) was used: obstructive CAD was defined as ≥50% of stenosis and nonobstructive CAD was defined as <50% stenosis, or normal coronary arteries, according to the prior studies [7]. A retrospective review of coronary angiography reports classified coronary arteries according to presence/absence of ≥50% stenosis as well as IRA and non-IRA.

### CMR Imaging Protocol, Acquisition, and Analysis

CMR was performed using a 3 Tesla MRI scanner (Ingenia, Philips Healthcare, Best, the Netherlands) 5 ± 2 days post-primary PCI. The CMR protocol included the following sequences: Balanced Turbo Filed Echo (BTFE), native modified look-locker inversion (MOLLI) recovery T1 mapping, and late gadolinium enhancement (LGE). To assess left ventricle (LV) systolic function, consecutive 8-mm short-axis images and 2-, 3-, and 4-chamber long axis images of the LV were acquired using a cine BTFE. Short-axis cine images were acquired covering the entire LV using retrospective gating. Two to three-fold accelerated parallel imaging (SENSE) was used to shorten the breath-hold. Three-ventricular short axis MOLLI images were acquired at the base, midpoint, and apex of the LV for native T1 evaluation. Native T1 was acquired using MOLLI 5s(3s)5s. Typical imaging parameters − field of view: 300 × 300 mm, slice thickness 10 mm, slice gap 5 mm, flip angle 20°, repetition time 2.14 msec, echo time 0.96 msec, matrix 142 × 142 pixels, actual experimental pixel size = 2.1 × 2.1 mm, interpolated reconstructed pixel size = 1.17 × 1.17 mm, SENSE factor 2, trigger delay end-diastole, inversion times ranging from 129 to 5,466 msec, 206 msec acquisition time for single image, MOLLI native T1 maps were generated from 3 BTFE images with variable inversion preparation time as previously described [8]. A total dose of 0.5 mmol/cc, 0.4 cc/kg gadoteric acid (Gadovist, Schering, France) was injected in a 2-phase protocol: 5 mL/sec gadoteric were infused as bolus pushed by a 15 cm^3^ saline at 5 mL/sec, and 15 s later, the remaining contrast dose followed by 20 cm^3^ saline were infused at a slower rate of 1 mL/sec. Ten minutes after contrast injection, short-axis and 2-, 3-, and 4-chamber inversion recovery LGE images were acquired with an inversion-recovery gradient-echo imaging sequence to evaluate focal myocardial fibrosis. The infarct size was determined by the percentage of myocardial LGE that was calculated using the standard 5SD method (Medis, Leiden, the Netherlands, version 7.6). LV ejection fraction and T1 maps were analyzed using a dedicated platform (IntelliSpace Portal, version 11, Philips Healthcare, the Netherlands) by two experienced readers (YB, OG) who were blinded to the clinical data of patients.

T1 maps were evaluated for technical image quality, excluding segments with breathing, motion, or adjacent extra-cardiac tissues-related artifacts. After visual assessment of confident maps, motion correction was performed if needed. Segmental T1 values were derived from short-axis T1 maps using a 16 segment AHA model in relation to three major coronary artery territories [9, 10]. Segment 17 was excluded from the analysis since it was not included in the apical T1 map (8). According to the standard AHA segmental myocardial definition, segments 1, 2, 7, 8, 13, and 14 were attributed to left anterior descending (LAD) territory; segments 3, 4, 9, 10, and 15 were attributed to right coronary artery (RCA) territory; and segments 5, 6, 11, 12, and 16 were attributed to left circumflex (LCX) territory [10]. In cases of dominant LCX, segments 5, 6, 11, 12, and 16 were considered RCA territory in the per-segment analysis.

### Statistical Analysis

All variables with a normal distribution are expressed as mean and standard deviation (SD). Native T1 values were presented as mean ± SD value per coronary territory (LAD, CX, RCA) and compared between the IRA, non-IRA obstructive (stented and not stented), and non-IRA nonobstructive coronary arteries. The mean native T1 value (per coronary territory) was tested with a one-way ANOVA test (“overall *p* value”), linear regression (“*p* value for trend”), and a *t* test to compare the obstructive and nonobstructive groups (“*p*” value). The specificity and sensitivity of the mean native T1 value for predicting obstructive non-IRA stenosis (for each vessel separately) were assessed using the C-statistic, equivalent to the area under the receiver-operating characteristic curve [11], to suggest a native T1 value threshold for predicting obstructive CAD. Per-vessel analysis clustered by patient ID was performed among LAD/RCA vessels and among LCX vessels, using a logistic regression model for the outcome of obstructive stenosis in one of the non-IRA vessels. In order to improve model convergence, the average T1 value was transformed with the following formula: (x-mean)/SD, yielding odds-ratios for a change in T1's SD [12]. Models were adjusted for age and infarct size (LGE %). All tests were conducted at a two-sided overall 5% significance level (α = 0.05). All analyses were performed using R software (R Development Core Team, version 4.0.3, Vienna, Austria).

## Results

### Baseline Characteristics

The study cohort included 60 STEMI patients with a total of 960 myocardial segments, out of which 107 segments (11%) were excluded due to artifacts (LAD territory; *n* = 11, LCX territory; *n* = 48, RCA territory; *n* = 48). Thus, 853 myocardial segments comprised the study group. The control group included a total of 336 myocardial segments, out of which 22 segments (6%) were excluded due to artifacts (LAD territory; *n* = 10, LCX territory; *n* = 6, RCA territory; *n* = 6). Baseline characteristics of the study cohort are presented in Table 1. The mean age was 59 years (range 34–79), and 92% were male. Anterior, inferior, and lateral STEMI were documented in 35, 17, and 8 patients, respectively. Five patients that presented with primary ventricular fibrillation underwent short cardiopulmonary resuscitation with electrical cardioversion before or during primary PCI*.* Average LV ejection fraction was 53 ± 11%. The average infarct size by LGE % of LV mass was 26 ± 12. LGE was present only in infarcted segments.

### Coronary Angiography and Primary PCI

Single-vessel obstructive CAD was documented in 31/60 patients; 30/31 underwent revascularization, while one distal LAD-IRA was treated only conservatively. Double-vessel obstructive CAD was documented in 20/60; of them, complete revascularization (IRA and non-IRA) was performed in 9/20. Triple-vessel obstructive CAD was documented in 9/60 of the patients; of them, complete revascularization (IRA and non-IRA) was performed in 1/9 patient. Thus, a total of 40/60 patients underwent complete revascularization. The decision to re-vascularize a non-IRA during the primary PCI was under the discretion of the performing invasive cardiologist. Staged PCI was scheduled and performed (several weeks following discharge) in the rest of the patients 19/60. No patients with ≥50% left main stenosis were present in the cohort; thus, the revascularization of the left main stem was not performed.

### Native T1 Mapping

**Fig. 1:**
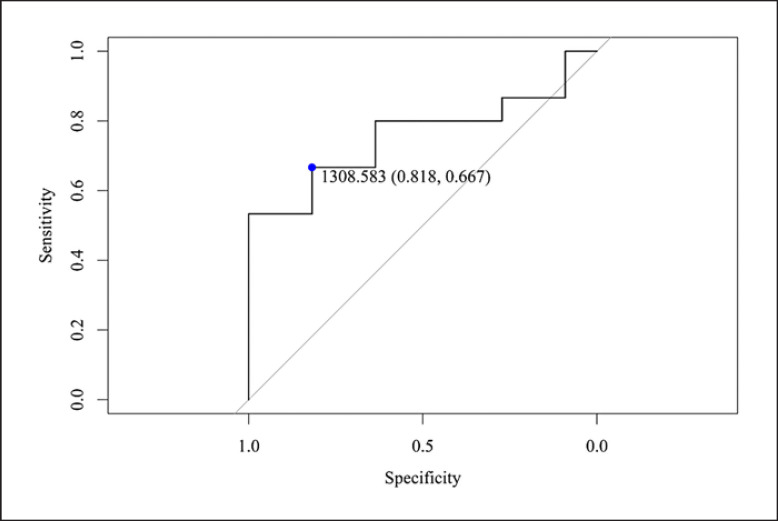
C-statistics for the LAD myocardial segments. An unadjusted logistic model was fitted with T1 average of the non-IRA for the LAD as a continuous parameter, and the outcome of any non-IRA LAD stenosis ≥50%; OR 1.03, 95% CI: 1.00–10.7. *p* = 0.04. The T1 threshold 1,309 msec demonstrated a sensitivity and specificity of 67% and 82%, respectively.

**Fig. 2:**
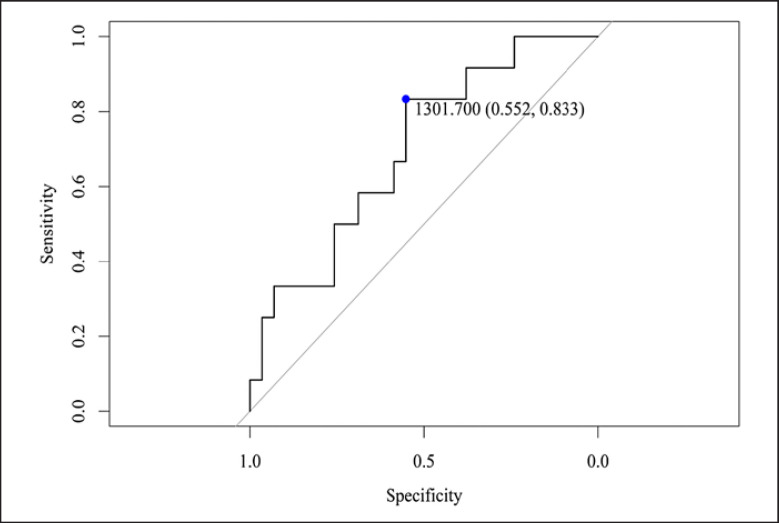
C-statistics for the RCA myocardial segments. An unadjusted logistic model was fitted with T1 average of the non-IRA for the RCA as a continuous parameter, and the outcome of any non-IRA RCA stenosis ≥50%; OR 1.02, 95% CI: 1.00–1.03. *p* = 0.05. The T1 thresholds of 1,302 msec demonstrated a sensitivity and specificity of 83% and 55%, respectively.

**Fig. 3:**
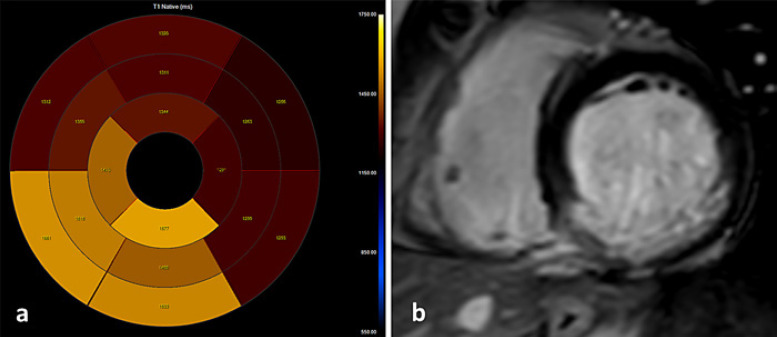
Clinical example: a 64-year-old male with inferior STEMI underwent primary PCI to the RCA. Concomitantly, a stenosis of 50–70% in the mid-LAD did not undergo PCI. **a** Native T1 map: elevated T1 values in RCA-IRA segments ranging from 1,460 to 1,550 msec and elevated T1 value in LAD segments ranging from 1,311 to 1,350 msec. **b** LGE in the short axis demonstrating subendocardial enhancement in the RCA territory.

**Fig. 4:**
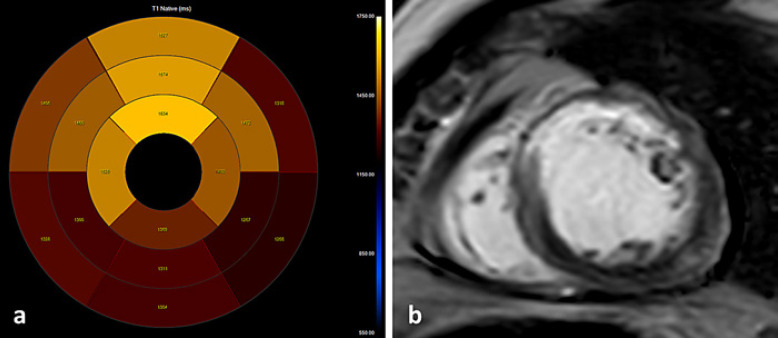
Clinical example: a 43-year-old male with anterior wall STEMI underwent primary PCI to LAD. Concomitantly, a dominant RCA with critical stenosis PCIed as well. **a** Native T1 map: elevated T1 values in LAD-IRA segments ranging from 1,406 to 1,634 msec and elevated T1 value in the RCA segments ranging from 1,304 to 1,369 msec. **b** LGE in the short axis demonstrating subendocardial enhancement in the LAD territory.

The mean native T1 values in the control group versus STEMI patients were 1,240.1 ± 81.4 and 1,362 ± 81.9 msec, respectively (*p* < 0.001). Native T1 values in all IRA territories were significantly higher in comparison to the non-IRA territory and the control group in a per-vessel analysis (Table 2). Native T1 values for the obstructive (≥50% stenosis) non-IRA were significantly higher in comparison to the nonobstructive non-IRA (<50% stenosis) for the LAD (*p* = 0.016) and RCA (*p* = 0.007) territories but not for LCX (Table 3). The cutoff native T1 value for predicting obstructive non-IRA LAD was 1,309 msec with a sensitivity and specificity of 67% and 82%, respectively (AUC 0.76, 95% CI: 0.57–0.95, *p* = 0.04), shown in Figure 1*.* The cutoff native T1 value for predicting obstructive non-IRA RCA was 1,302 msec with a sensitivity and specificity of 83% and 55%, respectively (AUC 0.7, 95% CI: 0.52–0.87, *p* = 0.05), shown in Figure 2. The cutoff native T1 value for predicting obstructive non-IRA LCX was 1,262 msec with sensitivity and specificity of 46% and 82%, respectively (AUC 0.6, 95% CI: 0.39–0.80, *p* = 0.31). Logistic regression model to predict the obstructive non-IRA after adjustment for age and infarct sizes demonstrated that native T1 value was an independent predictor of obstructive non-IRA LAD (OR 95% CI: 4.65, 1.32–26.96, *p* = 0.05) and obstructive non-IRA RCA (OR 95% CI: 3.70; 1.44–16.78, *p* = 0.03 but not obstructive non-IRA LCX (OR 95% CI: 0.67; 0.30–1.41, *p* = 0.44) (Table 4). Clinical examples including native T1 maps and short axis LGE images of 2 STEMI patients are presented in Figures 3 and 4.

## Discussion

The main finding of the present study is the documentation of significantly increased native T1 values in myocardial territories supplied by non-IRA with obstructive coronary disease (≥50% stenosis) on CMR performed early following STEMI. Native T1 values were found to be independent predictors of obstructive non-IRA LAD and RCA, after adjustment for the patient's age and infarct size. Using receiver-operating characteristic analysis, native T1 thresholds for distinguishing obstructive from nonobstructive vessel were found to be 1,309 msec for the LAD and 1,302 msec for the RCA. Thus, the early post-STEMI CMR with native T1 mapping may offer noninvasive, quantitative evaluation of the entire myocardium including the either infarct or the remote non-infarct territories. Despite the finding of significantly elevated T1 values in IRA-LCX, T1 values were not found to be significantly different between obstructive and nonobstructive non-IRA LCX. This was partly because of the cases of dominant LCX artery, LCX segments were classified as RCA, thus decreasing the number of the actually analyzed LCX segments, thus influencing the statistical results. Interestingly, no significant difference was found between native T1 values in the stented versus non-stented obstructive non-IRA in STEMI patients. A plausible explanation could be that early post-PCI myocardial relaxation time does not immediately normalize due to residual stunning or micro-embolization during PCI.

Native T1 values reflect the physical magnetic properties of myocardium, measuring the longitudinal relaxation time, and are increased in segments with myocardial impairment. Native T1 mapping allows the detection of focal or diffuse myocardial fibrosis in various conditions, e.g., cardiomyopathies, myocardial inflammation (as myocarditis) or infiltration (e.g., amyloidosis), as well as ischemic myocardial damage. The cutoff stenosis of >50% for obstructive CAD has been used in prior nuclear study [7]. Liu et al. [13] analyzed 10 patients with angiographically significant stenosis (>50%) in 1 coronary artery, who underwent CMR at 1.5-T adenosine stress/rest T1 mapping to illustrate its ability to distinguish between myocardial tissue abnormalities. Using this cutoff, they showed ability of T1 to discriminate between borderline or not obstructive coronary vessel. Coronary lesions causing ≥50% stenosis may result in elevated native T1 values in the corresponding myocardial segments since the large mesh of microvasculature distal to the stenosis (resistance vessels) compensatory dilates to keep total vascular resistance and normal stable blood flow [13, 14]. Consequently, the resting blood volume in these myocardial segments increases significantly. This autoregulatory phenomenon forms the basis for the detection of increased resting native T1 in the areas subtended by significant coronary artery stenosis.

Multivessel coronary disease is common among STEMI patients undergoing primary PCI and is associated with increased 30-day mortality [15]. Previously published magnetic resonance studies of STEMI patients showed that elevated native T1 values in remote (non-infarct) territories were independently associated with LV systolic dysfunction, worse prognosis, and increased infarct sizes [16, 17, 18, 19]. It was suggested that the remote tissue damage could be related to extracellular volume expansion and LV remodeling after STEMI [20, 21]. Functional recovery and chronic infarct sizes were also correlated with increased native T1 values and extracellular volume in the remote myocardium [22, 23]. It should be emphasized that no correlation was reported in these studies between LV dysfunction and worse prognosis with the presence of coronary stenosis other than IRA. To the best of our knowledge, our study is the first in STEMI patients to report the ability of elevated native T1 values to predict the obstructive non-IRA. Compared to previous studies based on 1.5 Tesla, the use of 3 Tesla scanner may improve image resolution and better discriminate between different coronary segments, thus explaining the ability to correlate increased T1 values and segmental coronary artery stenosis. The fact that there is a correlation between the presence of obstructive coronary artery and high native T1 values might support the performance of complete revascularization early after STEMI without postponing and waiting for the results of perfusion studies. As opposed to myocardial perfusion imaging, native T1 evaluation can be performed safely early post-STEMI, not exposing patients to pharmacologic or physiologic stress [2]. Additional studies are needed to assess the usefulness of revascularization of elevated T1 areas early after MI.

### Study Limitations

The study is retrospective with a relatively small cohort. There were a relatively small number of patients with LCX-IRA. Also, LCX segments were documented with non-negligible artifacts, leading to exclusion from analysis. Thus, the analysis of LCX-related myocardial segments was not representative. Native T1 values were not evaluated before and after revascularization, and thus, the discussion regarding the peri-procedural native T1 dynamics is beyond the scope of this study. Extracellular volume was not analyzed in infarct or non-infarct remote areas. It also must be mentioned as a clinical heterogeneity in the study by leaving complete revascularization at the discretion of the interventional cardiologist.

## Conclusion

The current study documented significantly higher mean native T1 values in the non-infarct territories supplied by obstructive coronary arteries. Elevated native T1 values are independent predictors of obstructive non-IRA in STEMI patients.

## Statement of Ethics

The study was reviewed and approved by the Sheba IRB-Helsinki Committee (International Review Board for Human and Animal Trials) 5897-19-SMC. Written informed consent for patient participation was not required for this study in accordance with the national legislation and the institutional requirements.

## Funding Sources

No funding sources to declare.

## Authors Contribution

Yafim Brodov: substantial contributions to the conception or design of the work; acquisition, analysis, gathering or interpretation of data for the work; drafting the work and revising it critically for important intellectual content; and final approval of the version to be published. Shlomi Matetzky and Eli Konen: drafting the work or revising it critically for important intellectual content and final approval of the version to be published. Mattia Di Segni: contributions to the design of the work (data gathering). Anan Younis: contributions to the design of the work (data gathering and management). Ayas Massalha, Anat Berkovitch, and Fernando Chernomordik: contributions to the design of the work (data management). Arkadi Beytelman: contributions to the design of the work (CMR technical support). Orly Goitein: substantial contributions to the conception or design of the work; acquisition, analysis, or interpretation of data for the work; drafting the work or revising it critically for important intellectual content; and final approval of the version to be published.

## Conflict of Interest Statement

No conflict of interest to disclose.

## Data Availability Statement

All data generated or analyzed during this study are included in this article. Further inquiries can be directed to the corresponding author.

## Figures and Tables

**Table 1:** Baseline characteristics of STEMI cohort

Variables	Patients
Age, years	59±10
Male	55 (92%)
Smoking	20 (33%)
Hypertension	20 (33%)
Diabetes mellitus	14 (23%)
Dyslipidemia	47 (78%)
Family history of ischemic heart disease	16 (27%)
LAD-IRA	35 (59%)
LCX-IRA	8 (13%)
RCA-IRA	17 (28%)
Peak troponin I, mg/dL	43±25
Cardiopulmonary resuscitation	5 (8%)
Left ventricular ejection fraction, % (range)	53±11 (34–78)
Infarct size by LGE, % of LV mass (range)	26±12 (7–50)

Categorical variables are expressed as number (percentage), continuous variables are expressed as mean ± SD. LAD, left anterior descending; LCX, left circumflex; RCA, right coronary artery; IRA, infarct related artery; LGE, late gadolinium enhancement; LV, left ventricle.

**Table 2:** Mean native T1 values in IRA and non-IRA territories supplied by obstructive (≥50% stenosis) and nonobstructive (<50% stenosis) coronary arteries

Coronary artery (*n* = 60)	IRA	Non-IRA ≥50% stenosis, stented	Non-IRA ≥50% stenosis, not stented	Non-IRA <50% stenosis	Control group (*n* = 21)	value overall	*p* value for trend
LAD	1,459±70, *n* = 34	1,334±7.5, *n* = 2	1,319±43, *n* = 13	1,283±32, *n* = 11	1,253±39	<0.001	<0.001
LCX^a^	1,338±71, *n* = 9	1,236±13, *n* = 2	1,286±51, *n* = 10	1,293±42, *n* = 39	1,233±44	0.03	0.14
RCA	1,438±92, *n* = 17	1,374±101, *n* = 3	1,334±43, *n* = 9	1,295±36, *n* = 31	1,224±63	<0.001	<0.001

All variables presented as mean ± SD. IRA, infarct related artery; LAD, left anterior descending; LCX, left circumflex; RCA, right coronary artery.

a Among dominant LCX arteries: IRA (*N* = 4), non-IRA with <50% stenosis (*N* = 5), ≥50% non-IRA (*N* = 0).

**Table 3:** Mean native T1 values in non-IRA territories supplied by obstructive (≥50% stenosis) and nonobstructive (<50% stenosis) coronary arteries

Non-IRA	Non-IRA ≥50% stenosis, stented/not stented	Non-IRA <50% stenosis	*p* value
LAD (*n* = 25)	*n* = 14	1,321±40	*n* = 11	1,283±32	0.016
LCX (*n* = 52)	*n* = 13	1,279±51	*n* = 39	1,293±42	0.31
RCA (*n* = 43)	*n* = 12	1,345±61	*n* = 31	1,294±36	0.007

All variables presented as mean ± SD. IRA, infarct related artery; LAD, left anterior descending; LCX, left circumflex; RCA, right coronary artery.

**Table 4:** Logistic regression model for predicting non-IRA obstructive CAD (≥50% stenosis)

Variable	LAD OR (95% CI)	*p* value	LCX OR (95% CI)	*p* value	RCA OR (95% CI)	*p* value
Age (years)	0.98 (0.88–1.08)	0.64	1.05 (0.98–1.13)	0.19	0.95 (0.87–1.03)	0.21
Infarct size (%)	0.96 (0.89–1.04)	0.33	0.98 (0.92–1.03)	0.32	0.93 (0.85–1.01)	0.09
T1 (mean ± SD)	4.65 (1.32–26.96)	0.05	0.67 (0.30–1.41)	0.31	3.70 (1.44–16.35)	0.03

IRA, infarct related artery; CAD, coronary artery disease; OR, odds ratio; CI, confidence interval; LAD, left anterior descending; LCX, left circumflex; RCA, right coronary artery; SD, standard deviation.
